# Multi-IRS-Assisted mmWave UAV-BS Network for Coverage Extension

**DOI:** 10.3390/s24062006

**Published:** 2024-03-21

**Authors:** Sota Yamamoto, Jin Nakazato, Gia Khanh Tran

**Affiliations:** 1 Department of Electrical and Electronic Engineering, School of Engineering, Tokyo Institute of Technology, 2-12-1 Ookayama, Meguro-ku, Tokyo 152-8552, Japan; yamamoto.s.bl@m.titech.ac.jp; 2 Graduate School of Information Science and Technology, The University of Tokyo, 7-3-1 Hongo, Bunkyo-ku, Tokyo 113-8654, Japan; jin-nakazato@g.ecc.u-tokyo.ac.jp

**Keywords:** Industry 5.0, digital twin, millimeter wave, UAV cellular networks, IRS, coverage extension

## Abstract

In the era of Industry 5.0, advanced technologies like artificial intelligence (AI), robotics, big data, and the Internet of Things (IoT) offer promising avenues for economic growth and solutions to societal challenges. Digital twin technology is important for real-time three-dimensional space reproduction in this transition, and unmanned aerial vehicles (UAVs) can support it. While recent studies have explored the potential applications of UAVs in nonterrestrial networks (NTNs), bandwidth limitations have restricted their utility. This paper addresses these constraints by integrating millimeter wave (mmWave) technology into UAV networks for high-definition video transmission. Specifically, we focus on coordinating intelligent reflective surfaces (IRSs) and UAV networks to extend coverage while maintaining virtual line-of-sight (LoS) conditions essential for mmWave communication. We present a novel approach for integrating IRS into Beyond 5G/6G networks to enhance high-speed communication coverage. Our proposed IRS selection method ensures optimal communication paths between UAVs and user equipment (UE). We perform numerical analysis in a realistically modeled 3D urban environment to validate our approach. Our results demonstrate significant improvements in the received SNR for multiple UEs upon the introduction of IRSs, and they confirm the feasibility of coverage extension in mmWave UAV networks.

## 1. Introduction

Recently, Industry 5.0 has been envisioned by the European Commission as a road map for the future, thereby aiming to cultivate a prosperous society through the strategic use of advanced technologies such as artificial intelligence (AI), robotics, big data, and the Internet of Things (IoT) [1]. These technologies find practical applications in digital twins (DTs), which serve as the conduit between the real and virtual worlds [2]. On the other hand, in recent years, with the improvement of hovering position accuracy and the update of control functions considering safety [3], unmanned aerial vehicles (UAVs) are planned to be deployed in various fields such as agriculture, defense, delivery, and surveying, which will facilitate the realization of Industry 5.0 [4,5,6,7]. Furthermore, current research has shed light on the capabilities of nonterrestrial networks (NTNs) to equip UAVs and low Earth orbit satellites with robust communication systems. These networks are particularly valuable when terrestrial infrastructure compromises natural disasters such as earthquakes, lightning, and typhoons [8]. Among the promising solutions in this arena are UAV base stations (UAV-BS) [9]. Thanks to this feature, we can build temporary networks as wide as required and when we need them, thus making them not costly. These stations are increasingly seen as vital for enhancing communication performance in areas experiencing sudden spikes in data traffic or regions affected by disasters [10].

Research on UAV-BS began in the mid-2010s, and nowadays, UAV relay and base station demonstrations are being conducted by companies, albeit in limited scenarios. However, it has not yet reached the stage of practical operation. The main reason is that we are still exploring unique issues and solutions for airborne base stations, which differ from existing base station designs. In addition, telecommunication organizations should standardize dissemination to many people, and governments should establish laws.

As in previous UAV network studies, Rahman et al. [11] proposed a centralized algorithm for positioning UAVs to maximize the throughput. Shen et al. [12] optimized the UAV’s moving trajectory for a UAV-enabled mobile relaying system. Vishnoi et al. [13] improved the energy efficiency of an entire intelligent reflective surface (IRS)-mounted UAV network. However, many studies on UAV networks employ microwaves, which limit the range of possible applications due to bandwidth constraints. There is also a pressing concern about frequency resource depletion, thereby necessitating new exploration into frequency sharing and developing higher frequency bands. To further the capabilities of UAV networks, especially for applications requiring high-speed and high-definition video transmission, we propose the utilization of the millimeter wave (mmWave) [14]. Using mmWaves also allows array antennas to be deployed on a UAV by making individual antenna sizes smaller. Therefore, the beam shape can be changed to suit the situation. Unlike traditional ground base stations, UAV-BS can be flexibly positioned in three dimensions, thus ensuring line-of-sight (LoS) communication, which is crucial for mmWave frequencies. This flexibility reduces signal attenuation caused by obstacles such as buildings. However, maintaining LoS paths to all user equipment (UE) for high-speed communication remains challenging in densely constructed urban environments.

On the other hand, NTT DOCOMO released the white paper for the 6th generation (6G) mobile communication system and stated a new radio network topology that increases the number of connection paths to the network [15]. It says that the IRS is an important technology. According to [16,17], the IRS is composed of a large number of elements that scatter electromagnetic waves, and it often uses metamaterial/metasurface technology that enables the design and control of the distribution of scattering properties over an area. Metasurfaces are thin sheets that can be fabricated as flexible sheets depending on the choice of base material, thus allowing them to be installed along the structure. This allows for control of radio waves’ scattering properties while maintaining the structure’s existing geometry. In general, the IRS is expected to adaptively control the radio environment by periodically repeating the following two actions. The first action is estimating the radio channel state information necessary to determine the IRS operation. The second one is controlling the scattering intensity and phase distribution on the IRS surface based on the estimated information to obtain the desired propagation channel.

This is the first paper to integrate the IRS into UAV-BS networks for coverage extension. Specifically, it applies realistic space–time UE distributions extracted from field data and reproduces existing building structures into our simulation environment for strictly judging blocking conditions between the transmitter and receiver for the sake of enhancing reality. The contributions of this paper are as follows:

- We propose a system model for multi-IRS-assisted UAV-BS networks to extend the coverage of mmWave communications.
- We construct a three-dimensional environment based on actual field data and evaluate the proposed system through numerical analysis.

The rest of this paper is structured as follows. Section 2 introduces previous research on the IRS. Section 3 states the overall architecture of the proposed system and the system model. Section 4 describes the proposed IRS selection algorithm with the actual environment. Section 5 presents numerical analysis results and discussions. Finally, Section 6 summarizes this paper and mentions future works.

## 2. Related Work

This section introduces the related research on the IRS from three points of view to highlight their differences from this paper. Table 1 summarizes all related works mentioned in this section. The IRS and RIS are treated as the same.

### 2.1. IRS Only or Integrated to BS

Some researchers investigated principles and recent trends in the RIS [18,19,20]. Muhammad et al. [18] described why the IRS is considered to play an important role in the next generation networks and introduced how people develop the IRS from electronics, mechanics, and materials approaches. Yuanwei et al. [19] described the basic principles of RISs both from physics and communications perspectives, based on which they presented the performance evaluation of multiantenna-assisted RIS systems. This study suggested that there are a lot of applications for the IRS as well. Although researchers normally assume that the IRS has prior knowledge of the propagation environment, Alamzadeh et al. [20] proposed IRSs with integrated sensing capabilities and designed IRSs electromagnetically. In ref. [21], the simulation results illustrate the importance of BS–RIS–user association optimization in RIS-assisted cellular networks and verify the effectiveness of the proposed joint association and beamforming design algorithm. Commercialization is also in progress. TMYTEK [22] developed an IRS product that has fixed angles of incidence and reflection of radio waves, which contributes to expanding indoor coverage by reflecting radio waves entering through windows. NTT DOCOMO created a prototype of a metasurface that can arbitrarily control the ratio of reflection and transparency of radio waves [16]. This design helps to seamlessly integrate with metropolitan building environments through the use of such transparent metasurfaces.

### 2.2. IRS on UAV

Both the IRS and the UAV are highly compatible with today’s advanced technology and are increasingly expected to be utilized in the future. Equipping the UAV with an IRS allows for more flexible deployment in three dimensions compared to conventional deployment on a building side. Rahmatov et al. [23] compiled the advantages and previous research of an RIS-carried UAV for 6G and mentioned that power consumption, channel estimation, the physical shape of the RIS, environmental factors like wind, and eavesdropper access can be issues. Many researchers are working on the above issues. Some use deep learning. Heejae et al. [24] formulated an RIS-carried UAV federated learning model, proposed resource allocation in an RIS controller, and achieved a maximum data rate. Kefeng et al. [25] showed that the RIS can significantly improve the system sum rate in the added signal reflection mode. Wen et al. [26] designed a new multifunctional RIS architecture and contributed to combating eavesdropping. A multifunctional RIS can simultaneously reflect and amplify the desired signal and emit friendly jamming. Xin et al. [27] accomplished a higher average downlink throughput by jointly optimizing the UAV trajectory, RIS passive beamforming, and source power allocation for each time slot. They indicated the effect of RIS–UAV relaying communication and the possibility of mounting many elements consisting of IRSs on UAVs.

### 2.3. IRS Collaborated with UAV-BS

The UAV functions as a BS/RS and communicates with the IRS deployed on the side of buildings in this related research. Ankur et al. [28] proposed an RISs selection scheme based on the composite UAV–RIS–user channel gains with the possibility of an imperfect and outdated CSI of the composite links. It has also been deduced from the results that it is always better to select fewer large-sized RISs than more small-sized RISs for a fixed number of total reflecting elements in the network. They used multiple RISs; however, the composite links were considered to be the LoS. The possibility that some paths may be NLoS should also be considered. Mohammad et al. [29] and Liang et al. [30] tried to analyze multihop RIS-assisted UAV communication’s outage probability, bit error rate, and SNR. They made links based on the Nakagami-m distribution or statistical distribution. As the frequency increases, whether the link is LoS or NLoS by obstructions becomes a significant issue. Therefore, it is necessary to construct a real-based simulation environment with buildings and judge the visibility instead of setting it probabilistically. Some researchers have taken advantage of the RIS-assisted UAV for IoT networks. UAVs collaborated with IRSs to overcome time and battery constraints when collecting data from sensing devices in Ref. [31]. RIS-assisted UAV communications were found to help wireless power transfer for many IoT devices, because UAVs have agility and can easily be recharged at the docking station [32]. Kefeng et al. [33] worked on a more extended RIS-assisted UAV network, which integrated satellite–UAV–terrestrial networks. Here, the IRS played an amplified-and-forward (AF) role, and the UAV functioned as a decode-and-forward (DF). Kefeng et al. [33] showed that the heavy channel fading, fewer signals, larger interferences, and larger impairment levels would bring worse system performance. Some researchers have aimed to minimize power consumption and maximize system energy efficiency [34,35,36]. But, in these research endeavors, one RIS was used, and the position of the UAV or IRS was fixed.

## 3. Architecture and System Model

This section introduces the architecture overview of our research and the access link system model focused in this paper.

### 3.1. Architecture

**Figure 1:**
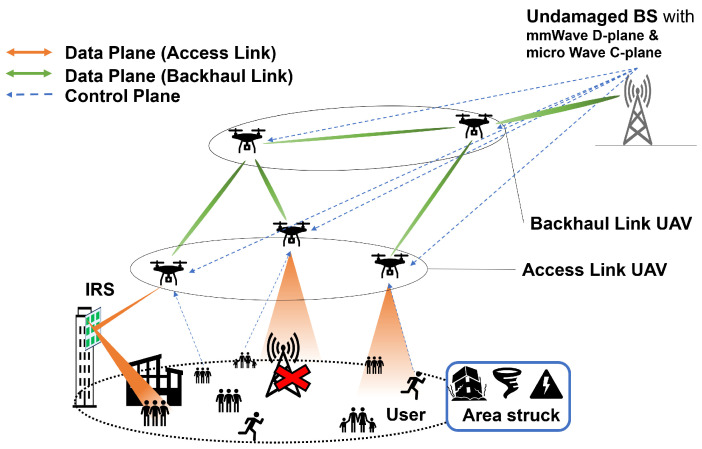
Proposed architecture overview.

Figure 1 presents the overall system architecture this paper aims to develop. The depicted scenario assumes that all BSs in a cell are nonoperational due to a disaster such as an earthquake, hurricane, or lightning, thus necessitating the establishment of temporary communication areas. As discussed in the previous section, UAVs offer a readily deployable solution. However, using UAVs merely as access points for end-users would limit their utility to the communication range of the BS. To overcome this limitation, the role of UAVs as relay stations becomes essential.

The figure illustrates two types of UAVs: access link UAVs, which provide an access link for end-user UEs, and backhaul link UAVs, which play as relay stations and facilitate backhaul connections between the access UAVs and neighboring undamaged BS. When access and backhaul UAVs operate autonomously, challenges arise, such as managing radio interference, ensuring adequate throughput, and avoiding redundancy of relay stations. To address these issues, coordinated operations of multiple UAVs become critical and require meticulous management and control. This is achieved by connecting all UAVs to the BS via a control plane. Small amounts of data, such as where the UEs are now, are also shared by using this control plane.

Our primary goal is extending the access UAV coverage area. To transmit detailed damage information to victims and rescue workers, access UAVs should be capable of high-capacity communications to all the UEs in the disaster area. mmWave has the potential for high-capacity communication due to its wide bandwidth but suffers from severe propagation attenuation due to blocking. Therefore, in this paper, we consider reflecting and delivering radio waves using the IRS existing in the city. We create a virtual LoS from the access UAV through the IRS to improve the received SNR even for UEs with Non-LoS (NLoS). In particular, we discuss the creation of UE distributions based on real-world data, thus constructing a simulation environment modeled after a real city, determining communication paths using IRS, and optimizing the UAV three-dimensional location. Finally, the effectiveness of the proposed method is verified by numerical analysis.

### 3.2. System Model

**Figure 2:**
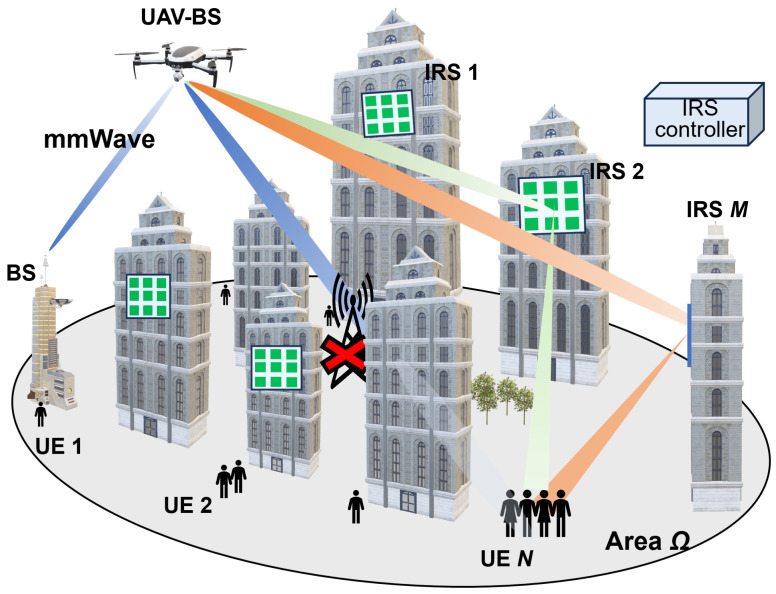
System model of IRS-assisted UAV networks.

Figure 2 presents the system model, thus illustrating how the access UAV extends coverage by selectively pairing one IRS with one UE from among multiple IRSs installed on structures such as buildings. This is how the UAV-BS creates a virtual LoS via an IRS for users located behind buildings who cannot obtain the LoS with a UAV. In this figure, ground BS is not available, and there are several possible routes for supplying radio waves from the UAV-BS. *N* denotes the total number of UEs in the specified area Ω, and *M* denotes the total number of IRSs. The position of neighboring BSs, UAVs, UEs, IRSs are respectively represented by the 3-dimensional coordinates $P_{\mathrm{BS}}\in R^{1\times 3},P_{\mathrm{UAV}}\in R^{1\times 3},P_{\mathrm{UE}}\in R^{N\times 3},$ and $P_{\mathrm{IRS}}\in R^{M\times 3}.$ The horizontal positions of the UEs are located outdoors within the specified area Ω.

We use real-based population data, that is, mobile spatial statistics [37], which are population statistics generated using the NTT DOCOMO cell phone network mechanism. A compartment is approximately 250 m × 250 m and can capture the total population. Within a compartment, UEs are set with the sum of hot spots and uniform distribution. The location, radius, number of hotspots, and number of UEs to be accommodated are set randomly.

#### Path Loss Model and Calculation of UE Received SNR

The path loss model between the UAV in the sky and the UE on the ground is computed as the sum of the free space radio wave propagation loss and additional loss, which varies depending on whether the connection is LoS or NLoS by blocking [38]. Atmospheric absorption losses are not considered because they are negligible [39]. In our simulation, the path length from the UAV to the UE is up to 3.4 km. Atmospheric absorption will be maximally 3.4 dB [39] and included in the additional loss.

(1) $$L_{\mathrm{LoS}/\mathrm{NLoS}}\left[\mathrm{dB}\right]=L_{\mathrm{free}}+\eta_{\mathrm{LoS}/\mathrm{NLoS}}$$

where $L_{\mathrm{free}}$ denotes free space radio wave propagation loss, which is calculated by

(2) $$L_{\mathrm{free}\;}\left[\mathrm{dB}\right]=20\log d+20\log f+20\log\frac{4\pi }{c}$$

where *d* denotes the communication path length between the UAV and UE, *f* denotes the carrier frequency, and *c* stands for the speed of light. Based on the Friis transmission formula [40], the received power $P_{r}$ at the UE is calculated by

(3) $$P_{r}\left[\mathrm{dBm}\right]=P_{t}+G_{t}+G_{r}-L$$

where $P_{t}$ denotes the transmit power from the UAV antenna, $G_{t}$ denotes the gain of the UAV transmitting antenna, and $G_{r}$ signifies the gain of the UE receiving antenna. From the obtained received power $P_{r}$, the SNR γ is calculated by

(4) $$\gamma =\frac{P_{r}}{P_{n}}$$

where $P_{n}$ denotes the noise power, which is calculated by

(5) $$P_{n}=\sigma_{n}^{2}B$$

where $\sigma_{n}^{2}$ denotes the noise power density, and *B* denotes the system bandwidth.

## 4. Proposed IRS Selection and Access Link Connection Approach with 3D Simulation Environment

In this section, we describe the simulation environment modeled after an actual city and present an optimal method for IRS selection and how to establish access links in UAV networks.

**Figure 3:**
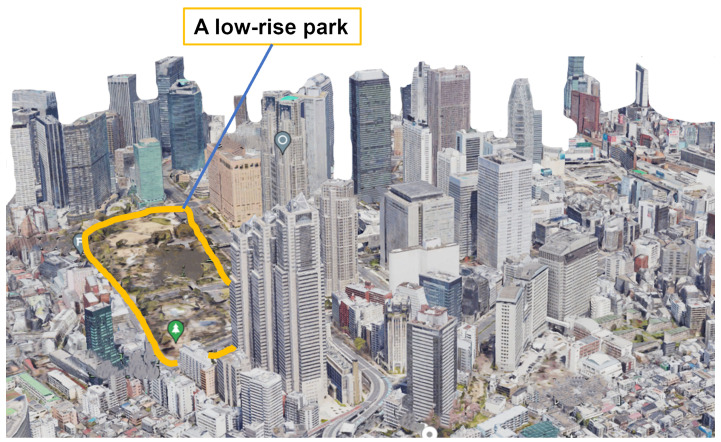
Actual environment map data around Tokyo Metropolitan Government Office in Google Earth.

**Figure 4:**
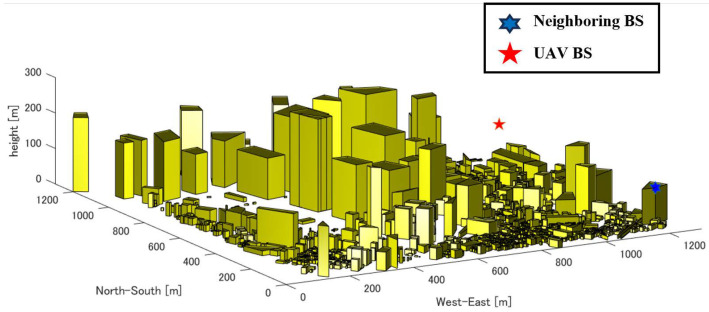
A 3D simulation environment around Tokyo Metropolitan Government Office.

**Figure 5:**
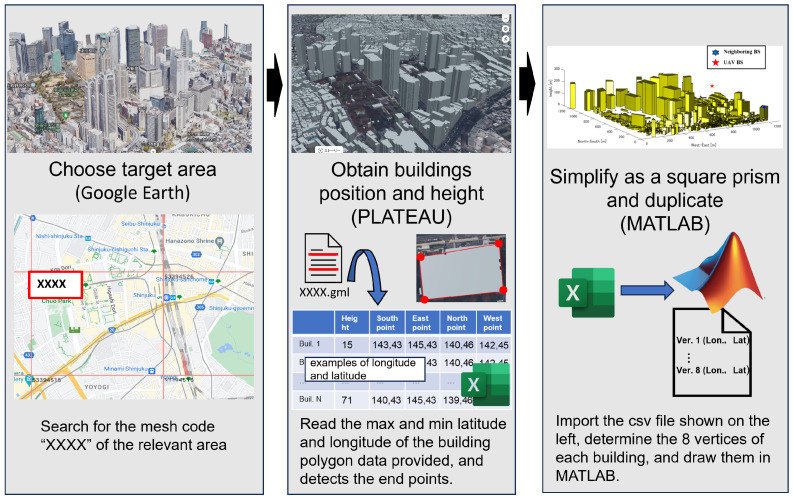
The sequence of replication.

Figure 3 shows the map data around the target area, while Figure 4 depicts the replicated simulation area. We sourced building locations and heights from PLATEAU [41], an open-data project led by Japan’s Ministry of Land, Infrastructure, Transport, and Tourism that is focused on 3D city modeling nationwide. The sequence of replication is shown in Figure 5. After reading the polygon data of a building and detecting endpoints based on the maximum and minimum values of latitude and longitude, we then simplified and replicated buildings as a quadrangle within MATLAB R2022a. In this way, an arbitrary city can be reproduced in MATLAB, and the necessary UAVs and IRSs can be retrofitted to achieve numerical analysis with a low processing load.

A LoS connection between the UAV and UE is highly likely in rural areas, where tall structures are sparse. In such cases, direct radio wave provision from the UAV is generally sufficient. Conversely, NLoS conditions between the UAV and UE become more probable in densely built urban environments with many tall buildings. This scenario increases the potential for improving UE reception power via IRS-assisted path modifications. The use of mmWaves allows for antenna miniaturization and the placement of multiple arrays. UAVs and IRSs can direct radio waves in any desired direction through beamforming (BF). mmWaves are characterized by their linearity and low diffraction, and they experience significant attenuation when encountering obstacles. So, it is not sufficient to make LoS judgments probabilistically as in the past; it is effective to make LoS judgments based on whether there is no obstruction between the sender and receiver. The high density of buildings also expands the number of potential sites for IRS installation. For this reason, we selected the Shinjuku Ward in Tokyo as our model environment. Shinjuku is renowned worldwide for its high-density urban layout. The specific area spans approximately 1.2 ${\mathrm{km}}^{2}$, with longitudes ranging from 139.6864° E to 139.7004° E and latitudes from 35.6829° N to 35.6943° N; it has a large park in the west and a cluster of high-rise buildings in the east.

### 4.1. Access Link Connection

As shown in Figure 4, some UEs may experience NLoS conditions with UAVs due to obstructions. This is particularly problematic for millimeter wave communication, where signal attenuation can be significant. Coordination with IRSs or the utilization of neighboring BSs is essential to address this issue.

Algorithm 1 determines the communication path from the UAV to each UE. It notices which UE can create an LoS. We assume that four IRSs are deployed on each side of a building and that there is a single neighboring BS. The determination of the access link communication path follows the procedures below. For a given UE, if the path between the UAV and the UE is in the LoS, direct communication is considered feasible. Otherwise, when it is in the NLoS, if the path from the UAV to the IRS is in the LoS, the incident angle of the radio wave at the IRS is within the threshold ζ, the path from the IRS to the UE is the LoS, and the reflection angle of the radio wave is within the threshold ζ; then, this UAV–IRS–UE link is established. If multiple IRSs satisfy these conditions, the shortest total path length is selected. Finally, if none of the above conditions are met, and the path from the neighboring BS to the UE is in the LoS, then this communication path is established. If the neighboring BS–UE link is in the NLoS, then the NLoS UAV–UE link is chosen.
**Algorithm 1** Decision of communication link between UAV and UE1:**Initialize:** The number of UEs *N*, The number of IRSs *M*, IRS angle threshold ζ2:**for** i=1,…N **do**3:    **if** UAV-UE(*i*) is LoS **then**4:        Connect UAV-UE(*i*) link directly5:    **else if** UAV-UE(*i*) is NLoS **then**6:        **for** j=1,…M **do**7:            **if** UAV-IRS(*j*) is LoS  Incident angle at IRS(*j*) ≤  ζ **then**8:                **if** IRS(*j*)-UE(*i*) is LoS  Reflection angle at IRS(*j*) ≤ ζ **then**9:                   Connect UAV-UE(*i*) link via IRS(*j*)10:               **end if**11:           **end if**12:       **end for**13:       When some IRSs satisfy the conditions, choose one that makes sum path length the shortest14:       **if** UE(*i*) does not associate with any IRSs **then**15:            **if** Neighboring BS-UE(*i*) is LoS **then**16:                Connect neighboring BS-UE(*i*)17:            **else if** Neighboring BS-UE(*i*) is NLoS **then**18:                Connect UAV-UE(*i*) link though it’s NLoS19:            **end if**20:        **end if**21:    **end if**22:**end for**

### 4.2. UAV Position Optimization

The UAV can flexibly change its position in three dimensions according to the environment due to its characteristics. In this study, a simulation is conducted considering a disaster area as a use case. In order to communicate detailed damage information to disaster victims and rescue workers, the access UAV should be capable of high-capacity communication to all UEs. Therefore, UAVs are deployed to maximize the minimum SNR among all UEs. Several candidate points are prepared equally within the area, and the UE received SNR is calculated at all candidate points. The optimization problem P1 is shown below.

$$\begin{array}{cc} P1:\; & \max_{P_{\mathrm{UAV}}}\min\gamma \\ s.t.\;C1:\; & P_{\mathrm{UAV}}\left(1,1:2\right)\subseteq \Omega \\ C2:\; & z_{\min}\leq P_{\mathrm{UAV}}\left(1,3\right)\leq z_{\max}\\ C3:\; & \mathrm{Neighboring}\;\mathrm{BS}\;–\;\mathrm{UAV}\;\mathrm{is}\;\mathrm{LoS} \end{array}$$

where C1 is the constraint on the UAV horizontal position, C2 pertains to the constraint on the UAV vertical position, and C3 ensures that there is a LoS connection between the UAV and the nearby base station for establishing a stable backhaul link.

## 5. Simulation Results and Discussion

A simulation environment was constructed using a real city as a model, and numerical analysis was performed in MATLAB. The parameters are shown in Table 2. We executed downlink communication from one access UAV to multiple UEs, and we considered a mmWave UAV–BS network in an environment where multiple IRSs were deployed, thus aiming at coverage expansion. The communication path between the transmitter and receiver at those higher frequencies in 5G is easily blocked due to the existence of obstacles or blockages [42]. In that case, IRSs are introduced to artificially create communication paths to avoid blocking. A nearby undamaged ground BS was installed at 139.68676° E, 35.69350° N on the roof of a 209-meter-high building located in the upper left corner of the environment, which simulates the Shinjuku Metropolitan Government Office area in Tokyo. This BS established a wireless backhaul link with the access UAV, which provided signals to all UEs scattered in the area Ω, thereby utilizing the appropriate IRS according to Algorithm 1. IRSs were deployed on all four sides of a building that met the specified height criteria κ for a total of *M* IRSs. We assumed that the power reflection by the IRS occurred only once and that the IRS had a passive BF and could arbitrarily change the direction of the reflected wave. Glass and wood typically have low reflectivity. Concrete and metal have high reflectivity, but for mmWaves, most of them are rough surfaces, so diffuse reflections occur, and the energy appears to be dispersed in multiple directions. Therefore, we assumed a reflection coefficient of 1 at the IRS, and there was no reflection assumed outside of the IRS. The UAV knows the area map and the position of the IRS in advance. In contrast, the position of the UE is communicated to the UAV via the uplink control plane and shared from the UAV to the IRS with the UAV position information. Time division multiaccess (TDMA) was employed for multiplexing multiple UEs, and one IRS was used for one UE in a given time slot. The numerical analysis program consisted of three main phases: tentative placement of buildings, the IRS and UEs, an overlap check between buildings and UEs, and an SNR calculation for each UE. Let *L* denote the number of buildings in the target area Ω and *P* denote the number of candidate UAV placement points. A UE is repositioned and checked *q* times until the overlap with all buildings is eliminated. *q* varies from UE to UE. For phase 3, we tried to connect all UEs to all IRSs and select the ones that matched the criteria according to Algorithm 1, which requires approximately N×M×P steps of computation. Overall, the computational complexity of this numerical analysis is on the order of $\left(L+M+N\right)+N\times M^{q}+N\times M\times P$. The computation on our computer—whose specs are a 12th Gen. CPU, an i7-12700 intel core, 2.1 GHz, and 16 GB RAM—took over 20 h.

**Figure 6:**
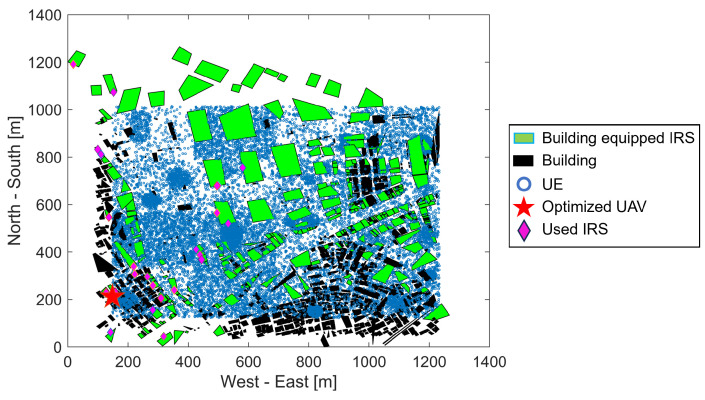
Position of UEs, buildings, IRSs, and UAV.

Figure 6 shows a two-dimensional plane illustrating the UAV placement and the IRSs used for path improvement for certain UE distributions. The horizontal axis represents the east–west distance in meters, while the vertical axis similarly indicates the north–south distance. Filled rectangles represent buildings, with those taller than κ and with an IRS-installed color-coded green, while all others were colored black. UEs depicted as blue circles are positioned so as not to overlap with buildings, and the red star indicates the optimal horizontal position of the UAV that maximizes the minimum reception SNR among all these UEs. IRSs that were used at least once to improve reception SNR are indicated with pink diamonds.

**Figure 7:**
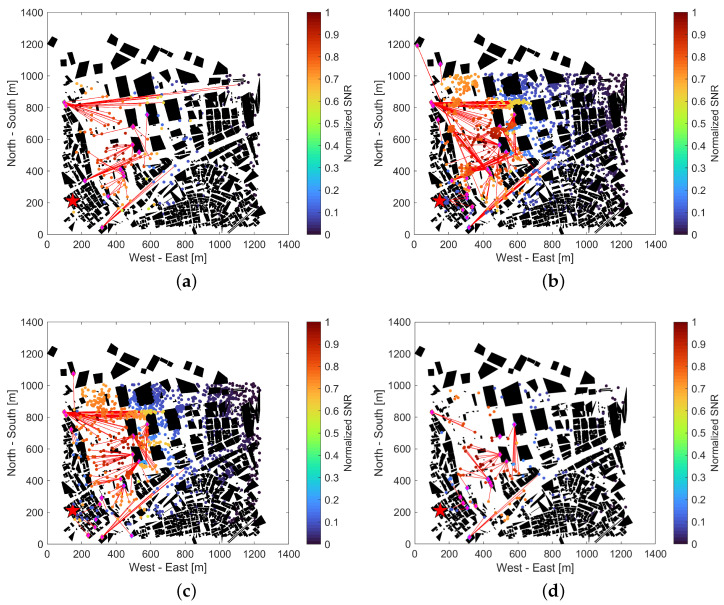
UE distribution and pairing with IRS on July 2024 weekday. (**a**) 6:00; (**b**) 12:00; (**c**) 18:00; (**d**) 0:00.

Figure 7 shows the pairs of IRSs and UEs used for route improvement connected by red lines for the distribution of UEs at 6:00, 12:00, 18:00, and 0:00 on a weekday in July 2024. The shown number of UEs is reduced to 1% of the true number of UEs in this figure just for the sake of visibility. Because the target area is a business area where the Tokyo Metropolitan Government and many companies set up their offices, the result indicates that the population is small in the early morning and late at night and increases during the daytime. It can also be confirmed that the number of IRSs utilized increases or decreases according to the population and distribution.

**Figure 8:**
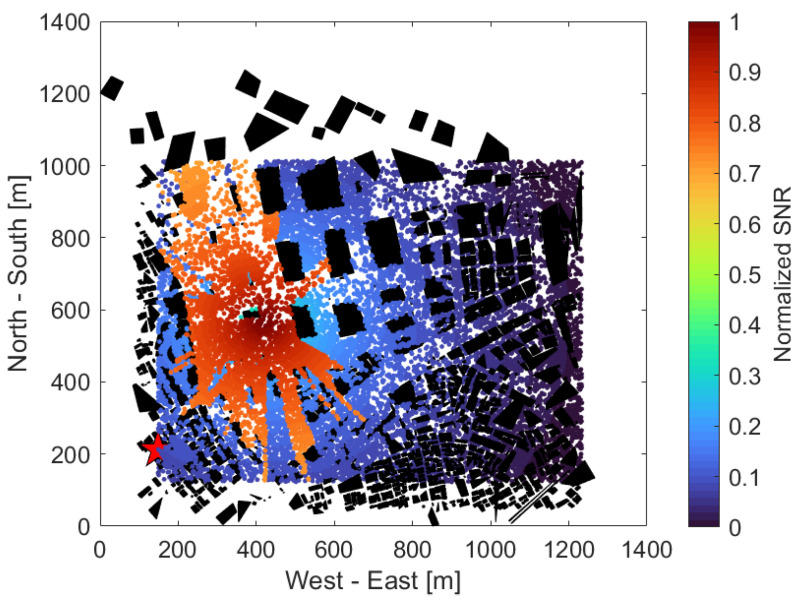
UE received SNR when not using IRS.

Figure 8 shows the received SNR of each UE without the use of an IRS, with the same UE distribution as in Figure 6. The horizontal and vertical axes are the same as in Figure 6. The color map on the right represents the normalized SNR, which has been normalized to a maximum value of 1 and a minimum value of 0 throughout all scenarios, regardless of the use of an IRS or adjacent BSs, with higher SNRs in red and lower SNRs in blue. From Figure 8, we can see that the SNR tended to be very high in the western part of the area near the UAV, that is, the part surrounded by (300, 300), (600, 400), (400, 1000), (200, 1000), and low in almost all other areas. The reason for this is a large park and a few tall buildings in the western part of the area, thus making it easier to create the LoS from the UAVs. On the other hand, the central area, for x axis values at approximately 600 to 800 and for y axis values at 500 to 1000, was lined with high-rise buildings, and the southeast and southwest areas surrounded by three points (600, 100), (1200, 600), (1200, 100) or (100, 100), (400, 100) (100, 800) were densely populated with small buildings, which may have prevented a clear LoS from the UAV and caused significant radio wave attenuation due to obstacles.

**Figure 9:**
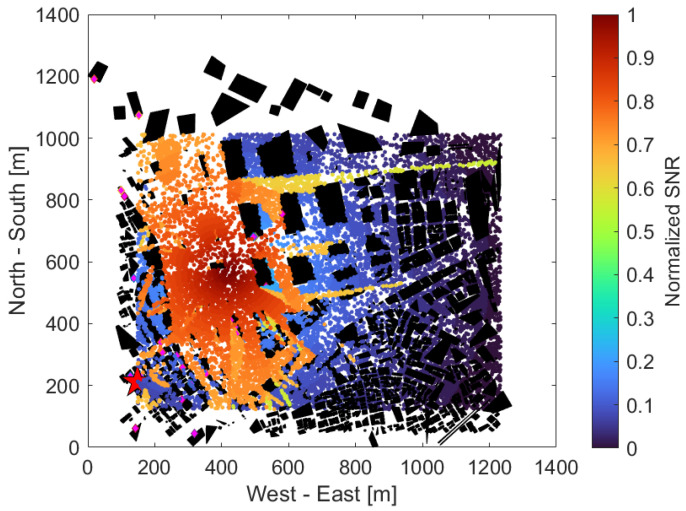
UE received SNR when using IRS.

Figure 9 shows the received SNR of each UE when using the IRS, with the same UE distribution as in Figure 6. Compared to Figure 8, the area with a higher SNR is spread over the southern and eastern parts of the park. The UEs located near the area’s northeastern edge (1200, 900) also provided a high SNR. This is thought to be because multiple IRSs around (300, 200), (400, 400), (500, 600), (100, 800) and so on were used to reflect the radio waves according to Figure 6 and establish a virtual LoS in the area that was shielded in Figure 8.

**Figure 10:**
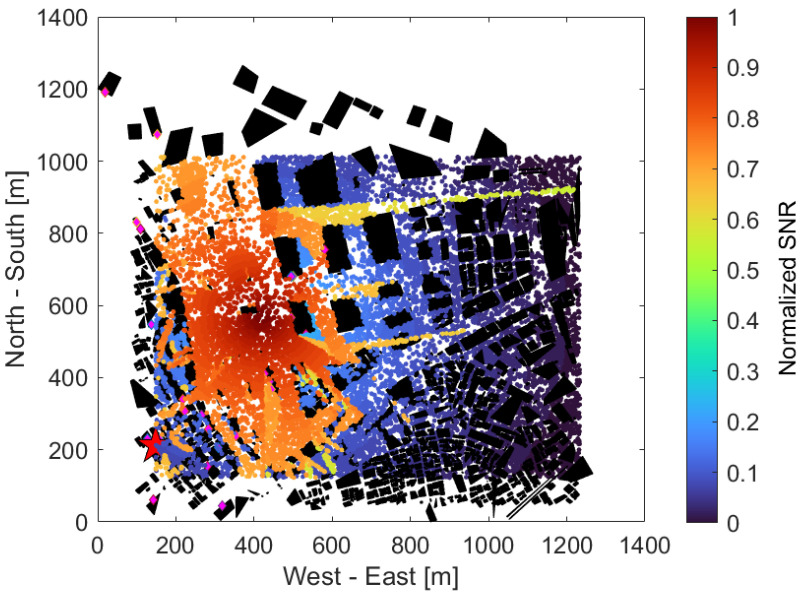
UE received SNR when using IRS & neighboring BS.

Figure 10 shows the received SNR of each UE in the situation shown in Figure 9, when it further utilized its neighboring BSs. Even if an UE could not establish an LoS with any IRS, it can establish a communication path by establishing an LoS with its neighboring BSs. As a result, it can be confirmed that the SNR was slightly improved around (x, y) = (200, 600) in the southwest part of the area compared to Figure 9.

**Figure 11:**
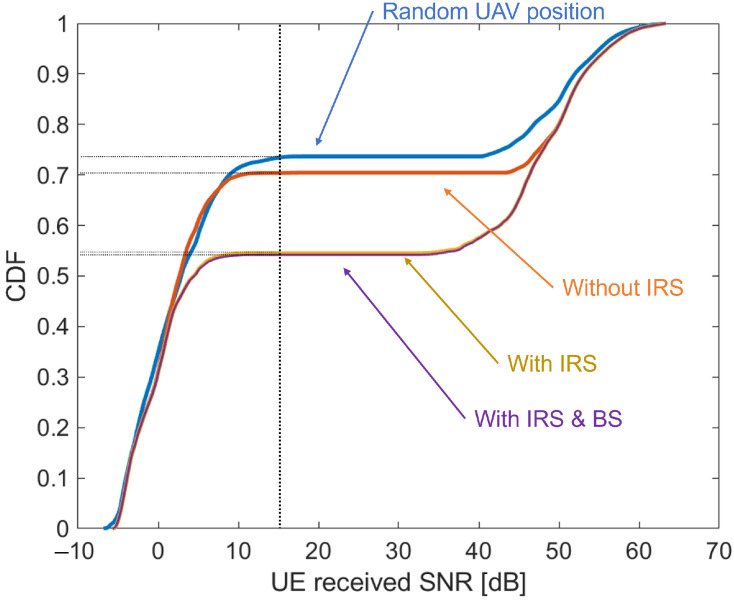
CDF of UE received SNR.

Figure 11 shows the cumulative density function (CDF) of the change in UE received SNR as visually confirmed in Figure 8, Figure 9 and Figure 10. The UE distribution was varied 10 times, and the results were tabulated. The horizontal axis represents the magnitude of the UE received SNR, and the vertical axis represents the CDF. The blue line indicates the case where the UAV position was randomly selected, the orange line indicates the case where the UAV position was optimized but an IRS was not used, the yellow line indicates the case where the UAV position was optimized and an IRS was used, and the purple line indicates the case where the UAV position was optimized and both an IRS and nearby BS were used. From Figure 11, the graphs changed significantly around SNR = 15 dB, which is considered to be the borderline between the NLoS and LoS in this simulation. Compared to the case where the access UAVs were placed randomly (blue line), the percentage of UEs that were NLoS decreased from 73.41% to 70.40% when deployed at the optimal location (orange line). In other words, 3% of the UEs were changed to the LoS, and the communication quality was improved. In addition, 16% of the UEs improved their communication quality by reflecting the radio waves through the IRS. Finally, for UEs that could not create a virtual LoS even with the IRS, we confirmed that providing radio waves from adjacent BSs improved the communication quality for 0.3% of the UEs. Although this value may seem small at first glance, it is expected to have a large impact depending on the simulation environment, since the adjacent BS was set to a specific location.

**Figure 12:**
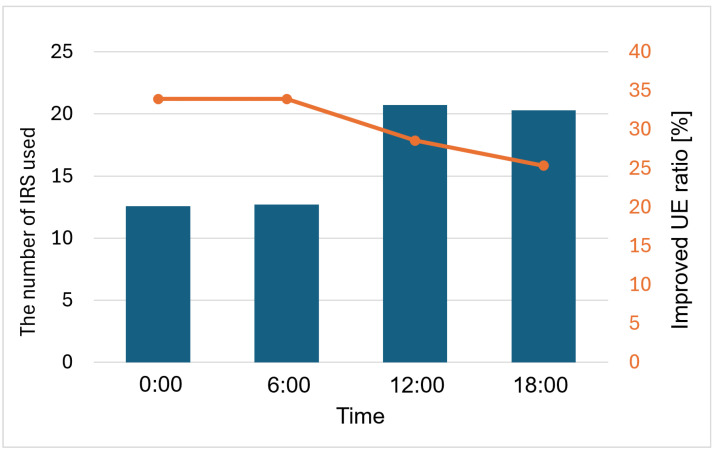
Time vs. Number of IRS used/SNR-improved UE ratio.

Figure 12 shows how many IRSs were used and how many UEs were improved for coverage extension at different times of 6:00, 12:00, 18:00, and 0:00. The bar chart shows how many IRSs were used at each time, thereby referring to the value on the left vertical axis. The line graph shows the percentage of UEs whose SNR was improved by the IRS relative to the total UEs in the area, and this refers to the value on the right vertical axis. To reduce computation time, the total population at each time was multiplied by a uniform constant. The results were averaged over seven simulations. The percentage of UEs with improved characteristics exceeded 33%, although the number of IRSs utilized in the late night and early morning hours when manpower is scarce was small. On the other hand, during the daytime and evening, when there is a large flow of people, a large number of IRSs were utilized in response to the increase in population, but the percentage of UEs with improved characteristics was 25~28%. It can be seen that there is a limit to the number of UEs whose characteristics can be improved from the limit of the number of IRSs installed in the area.

## 6. Conclusions and Future Work

This paper explored the potential for extending the coverage area of millimeter wave UAV-BS wireless networks by incorporating IRSs. We initially set forth distinct criteria for selecting the appropriate IRS and UE associations in an environment abundant with IRS installations. Subsequently, we proposed an algorithm to optimize the communication path between UAVs and UEs. Utilizing real-world field data as a basis, we modeled a three-dimensional environment in MATLAB and conducted numerical analysis to evaluate the effectiveness of our proposed algorithm. The results affirm that our algorithm, when used to integrate IRSs into UAV networks, enhances the received SNR for multiple UEs, thereby contributing to an expansion in a coverage area. Although the SNR at the physical layer was used as an evaluation index in this paper, throughput and other parameters at the upper layers can be calculated by introducing the network simulator ns3 [45]. Also, it will be possible to realize more complex and advanced simulations by setting up multiple access UAVs and designing multiplexing schemes, resource control, and cell partitioning among them.

As for future research, we aim to assess the quantitative effectiveness of our proposed algorithm in varied environments, including suburban and rural areas. Since this paper focused only on an ideal IRS functionality of being able to reflect radio waves in any direction, more practical IRS settings remain as our future works. It is also necessary to consider simulation with random nonfunctioning IRSs to imitate such scenarios when disaster occurs. Furthermore, incorporating different optimization methods for UAV positioning is expected to reduce computation time and help to determine the optimal location of the UAV more exactly.

## Figures and Tables

**Table 1:** Comparison of related technical works on several perspectives.

Aspect	Ref.	Main Contribution
IRS only or integrated to BS	[18,19,20]	Introduces what IRS is, its recent trends and future opportunities.
	[21]	Jointly optimizes BS–RIS–user association and beamforming design for cellular networks.
	[16,22]	Product commercialization efforts. Demonstration tests.
IRS on UAV	[23]	Compiles current research, challenges, and future trends on RIS-carried UAVs.
	[24,25]	Applies deep learning for RIS-assisted UAVs.
	[26]	Designs a new multifunctional RIS architecture.
	[27]	Jointly optimizes the UAV trajectory, RIS passive beamforming, and source power allocation.
IRS collaborated with UAV-BS	[28]	Proposes RIS selection scheme for UAV-based multi-RIS-aided multiuser.
	[29,30]	Presents a framework to examine the performance of multihop RIS-assisted UAV communication.
	[31,32]	Applies for IoT networks like timely data collection and wireless power transfer.
	[33]	Verifies satellite–UAV–terrestrial networks under the consideration of hardware impairments and interference.
	[34,35,36]	Jointly optimizes RIS phase shift and UAV position to improve power consumption.

**Table 2:** Simulation parameters.

Parameters	Value
Carrier Frequency *f*	28 GHz
Bandwidth	100 MHz [43]
Environment	Dense Urban, 139.6864° E ~139.7004° E,
	35.6829° N~35.6943° N,
	approx. 1.2 km × 1.2 km
UAV, UE area Ω	139.688° E~139.7° E,
	35.684° N~35.692° N,
	approx. 1.1 km × 0.9 km
UE height	1.2 m
IRS height κ	30 m
Range of incident and reflection angle at IRS ζ	15~75° (vertically, horizontally)
Neiboring BS position	(139.68676° E, 35.69350° N, 209 m)
Minimum UAV height	50 m
Maximum UAV height	70 m
The number of UAVs	1
The number of IRSs *M*	1000 (4 × 250)
The number of UEs *N*	13,349
The number of hotspots	0~3
The ratio of UEs inside hotspots	30~70%
Hotspots radius	10~20% of area length
EIRP (Trans. power + Antenna gain)	68 dBm [44]
Transmit output power $P_{t}$	45 dBm [44]
Tx antenna gain $G_{t}$	23 dBi
UE Rx antenna gain $G_{r}$	0 dBi
Noise density $\sigma_{n}^{2}$	−174 dBm/Hz
Additional loss by LoS $\eta_{\mathrm{LoS}}$	3.5 dB [38]
Additional loss by NLoS $\eta_{\mathrm{NLoS}}$	47 dB [38]

## Data Availability

Data are contained within the article.

## Abbreviations

The following abbreviations are used in this manuscript:

AI	Artificial Intelligence
UAV	Unmanned Aerial Vehicle
NTN	Nonterrestrial Network
mmWave	Millimeter Wave
IRS	Intelligent Reflective Surface
LoS	Line-of-Sight
UE	User Equipment
IoT	Internet of Things
BS	Base Station
AF	Amplified-and-Forward
DF	Decode-and-Forward
SNR	Signal to Noise Ratio
NLoS	Non-Line-of-Sight
BF	Beamforming
TDMA	Time Division Multiaccess
EIRP	Equivalent Isotropic Radiation Power
